# Trust and responsibility in food systems transformation. Engaging with Big Food: marriage or mirage?

**DOI:** 10.1136/bmjgh-2021-007350

**Published:** 2021-11-24

**Authors:** Joe Yates, Stuart Gillespie, Natalie Savona, Megan Deeney, Suneetha Kadiyala

**Affiliations:** 1 Faculty of Epidemiology and Public Health, London School of Hygiene and Tropical Medicine, London, UK; 2 International Food Policy Research Institute, Washington, District of Columbia, USA; 3 Faculty of Public Health and Policy, London School of Hygiene and Tropical Medicine, London, UK

**Keywords:** nutrition, public health, child health, health policy

## Abstract

Concentration of power among transnational ‘Big Food’ companies has contributed to food systems that are unsustainable, unhealthy and inequitable for people and planet. Given these commercial determinants of health, if ‘food systems transformation’ is to be authentic—more than a passing narrative—then leveraging Big Food is paramount. To this end, researchers, practitioners and policy-makers are increasingly encouraged to engage with these powerful entities. However, given the conflicts of interest at stake, engagement relies on trust and transparency, that all stakeholders take responsibility for their actions and demonstrate commitment to do no harm. Given Big Food’s track record in influencing policy, shifting costs and responsibility for their harms—and while profit primarily drives business decision making—we question whether it is logical to expect trust.

This analysis explores concepts of responsibility and trust in relation to food systems transformation involving public-private partnerships. Through short cautionary case studies—looking at the United Nations Food Systems Summit, and Big Food’s plastic burden—it argues that unless such companies take responsibility for their cross-cutting effects and earn authentic trust through demonstrably doing no harm, their participation in evidence generation and policy processes should be limited to responding to information requests and adhering to regulation. Any involvement in research agenda-setting or formulating policy solutions introduces conflicts of interest, legitimises corporate irresponsibility and jeopardises scientific integrity. Big Food has dynamism and power to address food system problems, but while it contributes to so many of these problems it should follow—not formulate—transformational evidence, policies and regulations.

**Summary box**

- Food systems are unsustainable, inequitable and unhealthy, with transnational ‘Big Food’ companies commercially determining the health of people and planet.
- Food systems transformation necessitates private sector engagement, but the private sector is diverse. The urgency and scale of food system problems requires change where the power lies: Big Food.
- Public–private partnerships require trust, which itself relies on responsibility, accountability and transparency.
- Given its track record, Big Food does not engender trust. Hence—while engagement should certainly take place (ie, through information sharing)—such companies should not be involved in agenda-setting.
- Inviting Big Food to join research and policy agenda-setting efforts legitimises irresponsible corporate behaviour and introduces significant conflicts of interest.

## Introduction

Food systems are broken and must be transformed is the narrative that has permeated global agendas and normalised into the mainstream. Such is its strength that the United Nations, backed by the World Economic Forum, held a Food Systems Summit—the first of its kind—in September 2021 to ‘launch bold new actions to transform the way the world produces and consumes food, delivering progress on all 17 Sustainable Development Goals.’^1^

Given the narrative, uncomfortable power-related questions must follow, such as: Who is responsible for these failures? Who should formulate and determine the solutions? What should these look like? How should they be implemented and monitored?

Many would point towards corporate social responsibility (CSR) and sustainability commitments of big business as proof of a willing and capable private sector best-placed to drive such transformation. Others perceive CSR initiatives as small-scale ‘side salads’ diverting attention from slow structural progress or business as usual.^2^ Centring the ‘private sector’ itself is problematic given its heterogeneity—encompassing not only large transnational companies but smallholder farmers and market traders. Disparities between these stakeholders are stark. On the one hand, are an estimated 2 billion people—many living under the poverty line—in smallholder (<2 hectare) farming households.^3^ On the other, is a small number of large transnational companies—predominantly based in high-income countries but with complex tax domiciles—concentrating power across food systems. This concentration is demonstrated by recent mergers in global seed and agrochemical sectors, where four companies—Bayer, Corteva, ChemChina-Syngenta and BASF—control 60%–80% of market share, stifle competition and foster interdependency between specific seeds, chemicals and technologies.^4^ The situation in global food and drink supply chains is similar,^5^ where such companies are often termed ‘Big Food’.

This unchecked concentration has permitted transnational companies to make exorbitant profits and exert undue policy influence, to the extent that regulations do not adequately protect against environmental, human health and social harms.^4 6^ In this sense, food systems—in their current form—may not be ‘broken’ but functioning as intended for those that prosper from the status quo.^7^

Therefore, if transformation is to actually be…transformative…for human and planetary health, change is needed where the greatest impacts, power and scale exist—in this case, among Big Food. However, the stimuli for such businesses to change their behaviour manifests in many forms, reflecting different worldviews—from ‘softer’ collaborative partnerships, self-regulation and shared goal-setting, to ‘harder’ fiscal and regulatory instruments. In other words, the much-fabled carrots and sticks.^8^ In a predominantly neoliberal global economic system—typified by revolving doors between industry and governments—softer approaches dominate.

It is within this context that researchers, practitioners and policy-makers generating and using multidisciplinary food systems evidence are encouraged to ‘engage with the private sector’. The rationale; that transformative change requires multistakeholder approaches with all actors around the table. The challenge though is that meaningful engagement requires trust. And trust relies on tenets such as responsibility and transparency. Both of which imbue power and are, therefore, often contested.^9^

This analysis explores concepts of trust and responsibility surrounding the types of Big Food companies that determine human and planetary health; companies increasingly engaged in codeveloping multistakeholder solutions for food systems transformation.^10^ We discuss themes relevant to this topic and present two short cautionary tales; one looking at Big Food’s involvement in the United Nations Food Systems Summit (UNFSS) and another on its plastic burden, in order to ask:

- What can history tell us about the willingness of transnational Big Food companies to take responsibility for, and act expeditiously on, their commercial determinants of public and planetary health?
- Given the conflicts of interest at stake, is it desirable or realistic to expect trust in Big Food, when the raison d'etre of such companies is to pursue profits, not social goods and public and planetary health?

## Context

### Are food systems broken?

Despite advances in productivity, food safety and supply chains, the prevailing ‘broken-needs-fixing’ narrative is hard to refute. Food systems account for a third of global greenhouse gas emissions^11^ and intensive industrial agriculture is depleting and damaging water, soils and forests rapidly.^12 13^ Meanwhile, supply-driven consumption within linear economies has flooded ecosystems with persistent organic pollutants and synthetic materials, including single-use plastics.^14 15^ Sustainably nourishing potentially 9 billion people within planetary boundaries is of urgent concern, particularly as a myriad of activities and populations comprising food systems are themselves exposed to the very environmental crises to which they contribute.^16 17^

Food systems are not just environmentally unsustainable, but unhealthy and inequitable—as evidenced by the ill affordability of healthy diets^18^ as well as uneven progress (and in some cases, regression) in all forms of malnutrition.^19^ This perfect storm has been called a global ‘syndemic’.^20^ Closely associated is the precarity of agricultural livelihoods—particularly of women and the marginalised—and the appropriation and destruction of Indigenous land, ways of life and knowledge systems.^21^ Taken together—and with the COVID-19 pandemic further exacerbating these frailties^19^—there is little doubt that food systems must change for people and planet. But what contributed to this calamity?

### Commercial determinants of planetary and population health

Commerce has affected human health ever since populations and cultures interacted, traded and subjugated one another.^22^ The impacts of consuming harmful commodities—particularly alcohol and tobacco—have been increasingly scrutinised and evidenced by public health communities.^23 24^ In recent years, evidence has accumulated on the multiple harms caused by consumption of ultraprocessed foods and sugar-sweetened beverages,^25 26^ and their increasing share in diets.^27^ However, it is not only the consumption of unhealthy products that can be harmful, indeed the production and supply of many products—both healthy and unhealthy—can cause broader harms. As such, ‘commercial determinants of health’ has emerged as a holistic framing of the human impacts of business activities across different sectors.^28^ This framing accounts for the practices, economic systems and regulatory environments that generate direct and indirect harms along supply chains and product life-cycles.^29^ A timely example of these life-cycle harms is the case of Tyson Foods—the USA’s biggest chicken processor—where deteriorations of working rights and conditions among farmers and employees is reported, alongside environmental and public health spillovers onto communities living nearby factories.^30^ Many of the problems associated with food systems are driven by the production and supply of foods that are artificially cheap because such environmental and social costs are not brought inside business practices or prices. Unhealthy commodity industries (UCIs) producing harmful products often share similar characteristics and maintain interwoven organisational relationships, employing—sometimes coordinated—approaches to influence policy and public discourse.^22 31^ Food systems are home to many such industries, where agro-chemical and agri-food companies interact and co-shape narratives,^32 33^ including recently to harness the COVID-19 pandemic through marketing strategies, political lobbying and CSR activities.^34^

Many Big Food companies, such as Bayer (pharmaceutical, agrochemical and life sciences), or Unilever (consumer goods), straddle different economic sectors and continents. These types of border-defying businesses generate profits and impacts across geographies and domains, affecting human health and the environment. Hence they are increasingly understood to be commercially determining planetary health.^35^ Such broader recognition signifies the importance of joined-up collaboration; between academic disciplines, government sectors and civil societies, to facilitate information sharing and action. But where does this leave the companies themselves? Can they be trusted to fix the problems they have created? Or is this akin to ‘putting Dracula in charge of the blood bank’?^36^

### Towards responsibility in the food system

Identifying commercial determinants of health is an important step towards accountability and evidence-based change, but is just that—a step. Transformation requires accountability that sticks; where stakeholders accept responsibility for harmful outcomes and comply with corrective and preventative policies (not to mention clean-up or compensation). However, apportioning accountability means weighing up social vs individual responsibilities; a process that reflects the spectrum of philosophical positions concerning how humans should or shouldn’t be governed and the degree to which free-market capitalism prevails.^22^

Where malnutrition is concerned, some state that ‘to understand who is responsible[…]it is first necessary to ask: Who rules global food systems?’.^33^ The answer is not consumers, whose choices—although complex and multifaceted—are confounded by an ‘illusion of diversity’ in markets dominated by a handful of companies promoting near-identical, cheap and unhealthy products.^37^ Choice is the point at which individuals interact with the food environment. It forms the bridge in their relationships with the architects of that environment; the food industry and government. The food industry provides choice; government shapes and regulates these choices (often under industry influence or civil society pressure); and individuals operate within the resulting space. In competing with industry-concocted psychological marketing campaigns, distraction techniques and political lobbying to deprioritise progressive diet-related policies, consumers’ ability to ‘vote with their feet’ may be something of a fallacy. Indeed, amid food systems so influenced by powerful interests, personal choice (*vis a vis* personal responsibility) is something of a misnomer. When consumer choice is genuinely possible, it is among a relatively privileged minority who are able to eat healthily, or to purchase fair or sustainably produced items. At national and supranational levels, governments and multilateral agencies are themselves apparently stunted in their ability to enact structurally transformative, cross-cutting and robust food systems regulation to protect people and planet. Whether it is failing to close tax avoidance loopholes, to limit volatile financial speculation, to enforce adequate anti-trust laws, or to truly protect human rights, policies affecting food systems appear at best to be ‘tinkering at the margins’^38^ and at worst facilitating Big Food’s priorities and concentration of power.

However, that is not to say the public and their elected representatives are destined to be passive bystanders in broken food systems. Quite the opposite. Social change has only ever been realised when civil society exerts a unified voice that governments cannot ignore; hence the growing calls for collective action and solidarity to challenge food systems power imbalances and realise the right to food.^39–41^ Discursive progress towards this can be found in language such as **‘**industrial epidemics’^42^ that—like ‘commercial determinants of health’—shifts the focus of responsibility from consumers back to producers and marketeers.

However, while responsibility may be attributed to Big Food, engaging such companies in research and policy processes to ‘transform’ food systems necessitates mutual trust; that stakeholders can be relied on to act with integrity and share common vision and commitment towards healthy humans and planet. Without this, meaningful engagement may be a mirage.

### Trust and engagement

Trust is an intangible construct with many definitions, ranging from the multidimensional (encompassing tenets such as competence, consistency and empathy) to the sociopsychological (such as the trust bestowed in institutional structures that regulate everyday life, or the calculative and rational trust mediated by logical assumptions and experiences).^43 44^ Since these definitions commonly overlap, what perhaps matters more is how trust can be built and maintained between Big Food, public researchers and policy-makers across government, given the scale and urgency of the global syndemic which necessitates leveraging and rebalancing of commercial power.

Cvitanovic *et al*^45^ present fourteen trust-building strategies along with a stepwise process for repairing damaged relationships at the interface of science and policy. At the very top of this list sits transparency; a fundamental characteristic acknowledged by others to be of foundational importance, particularly when combined with honesty and dignity.^44^ But while these characteristics may be crucial to public-private partnerships in general, where food systems are concerned, a consensus for pursuing engagement in the first place does not exist.

Stuckler and Nestle^33^ describe three stances on public health communities engaging with Big Food. First, a hands-off off approach that promotes self-regulation and leaves responsibility for healthy choices to the public; second a commitment to partnering with industry; and third, a sceptical position that refutes both other stances given their inherent conflicts of interest, instead advocating governmental assertiveness through regulation. A recent analysis among academic, business and public sector food system stakeholders suggests the third stance is a common barrier to public-private partnerships, not least because it involves trust, or lack thereof.^46^ This is perhaps unsurprising, since conflicts of interest—real or perceived—impact research agendas, scientific integrity and policy.^47 48^

However, where engagement may still be pursued, principles have been developed to aid careful navigation around conflicts of interest.^49 50^ One such tool, by Galea and McKee^51^ proposes five tests for public partnerships with large corporations. The first asking: are the ‘core products and services provided by the corporation health enhancing or health damaging’. This initial hurdle—viewed through a commercial determinants of health lens—would preclude partnership with many Big Food companies, even if their market concentration means they offer both healthy and unhealthy products. Unlike ultraprocessed foods, tobacco or alcohol, the human and planetary health characteristics of many individual products is often debatable or on a sliding scale based on consumption, thus complicating matters further.

These are the quagmires faced by public researchers, practitioners and government policy-makers.

## Cautionary case studies

Boxes 1 and 2 present two short case studies looking at the conduct of Big Food companies, illustrating the types of conduct and infiltration of agenda-setting processes that jeopardises trust among researchers, practitioners and government policy-makers.

Box 1 **Multistakeholderism in food system policy-making: the United Nations Food Systems Summit (UNFSS)**

In late July 2021, the UNFSS held a ‘Pre-Summit’—2 months before the main event took place in New York.

Many organisations and individuals engaged in the UNFSS consultative process via various online consultations since late 2020—while others boycotted it and organised a counter mobilisation.^53^ Critics argue that governance of the Summit process was opaque, principles of engagement with the private sector were weak, conflicts of interest and power asymmetries were not systematically acknowledged or addressed, and human rights were marginalised^54 55^ UNFSS leaders largely met this criticism with silence.

The Summit positioned itself as a ‘people’s summit’, with leaders highlighting the importance of participation. The widely espoused concept of ‘multistakeholderism’ opened the door to engagement with multinationals whose products and practices have been shown to drive malnutrition and planetary health harms. No representative of a multinational company sat on the formal leadership ‘action tracks’ but they actively participated in the Private Sector Guiding Group (PSGG). The PSGG was led by the World Business Council for Sustainable Development (WBCSD)—an association with an open membership, including tobacco giant Philip Morris and many multinational food companies whose products and practices have been associated with malnutrition (especially obesity and linked non-communicable diseases).^56^ Two of the latter—Nestlé and PepsiCo—were invited to speak at several Summit sessions.

The WBCSD ran other sessions including one in which a speaker from European Food Information Council (EUFIC) sowed doubt on the definition of, and harms caused by, ultraprocessed foods, despite the growing evidence of the latter (EUFIC count Coca Cola, Cargill and Bunge among their Board members.)

Publicly, the UNFSS positioned itself as a participation-based public–private partnership, while privately, the role of multinationals in decision making remained unclear throughout. But what was clear was the presence of Big Food around the high table where the shape of future food systems transformation was discussed and where decisions were made.

It is hardly surprising therefore that the Summit has been criticised for failing to acknowledge conflicts of interest. Its principles of engagement were weak—especially when compared with other UN conferences. They included vacuous ‘principles’ such as ‘be respectful’, ‘recognise complexity’ while omitting standard principles adopted by other UN processes such as ‘do no harm’. Complexity is often used by Big Food as a smokescreen to diminish the role of their products in poor health, for example, in the context of obesity by diverting attention away from harmful commodities towards physical activity.^57^

The final UNFSS principle was to ‘build trust’. Summit leaders often lamented a lack of trust in food systems governance. What was lacking however was a concerted attempt to uncover what drives this mistrust, and how it may be addressed. If this was another major systemic problem affecting food systems—for example, a lack of finance—there would have been working groups trying to figure out how to solve it. But nothing like that happened in the UNFSS process.

If we consider the trust-building strategies suggested by Cvitanovic *et al*^45^ in the context of the UNFSS, we hit the first stumbling block straight away: ‘ensure process transparency’. Transparency is a key component of good governance. As mentioned, many critics of the UNFSS process pointed to its opaque governance. Not much was publicly disclosed about the criteria for choosing action track leaders, the process for clustering and prioritising ‘game changing’ solutions, for deciding on commitments, and the accountability mechanisms for making any such commitments stick. Trust has to be earned and proactively built.

One of the most widely shared videos emerging from the UNFSS pre-summit was a 10 min speech by Professor Jeffrey Sachs in which he reminded participants that the most successful, evidence-based public–private partnership to date was…taxation: *‘To private sector leaders—behave, pay your taxes, follow the rules—that’s what you should do’.*

Box 2 **Where did they think it would go? The plastic burden of big food.**

Plastic pollution is a defining 21st century problem, increasingly recognised as a determinant of poor human and planetary health.^58^ This problem can no longer be ignored, hence a step-change in political and corporate action. But why did action take so long with evidence mounting for decades? One factor is (ir)responsibility.

Plastics are used extensively across food systems, particularly for packaging.^59^ A handful of companies dominate global food and beverage markets, including Coca-Cola, Nestlé, PepsiCo and Unilever. Together, these topped *Break Free from Plastic’s* 2020 brand pollution audit^60^ and generate an estimated 83 football pitches worth of unmanaged plastic waste each day.^61^

Such companies have set (and often missed) voluntary targets to increase recycled content of their products, while operating small-scale community corporate social responsibility projects that repurpose plastic waste.^62^ These strategies have thus failed on two major elements of the problem; to reduce virgin plastic use and prevent waste, at scale. Proven strategies, such as deposit-return schemes^63^ have been reluctantly agreed-to in some markets while actively fought against elsewhere.^64^ Rather than dent their operating profits (which, for the aforementioned companies exceeded $500B between 2010 and 2020) Big Food continued to prop-up the plastics industry and its Big Oil cousins through business as usual. Indeed it was only in 2019 that Coca Cola and PepsiCo—under pressure—departed the Plastics Industry Association, a major policy advocacy trade group.

By embedding themselves in overlapping webs of industry groups and arms-length initiatives Big Food has covertly lobbied governments and subtly shifted the discourse of responsibility so as to lumber the public with the costs of recycling.^62 64^ An infamous example is Coca Cola, which—alongside influencing public health policy in the USA^65^—finances *Keep America Beautiful*, the industry-backed group that produced the notorious ‘Crying Indian’ advert which emotively passed the buck of litter to the public by proclaiming: ‘people start pollution, people can stop it’.^66^ (infamous also because the actor playing the Indigenous man was actually Italian-American). But when waste management systems do not exist or cannot cope with demand, individual efforts are severely limited, hence the pandemic of ‘wishcycling’ wherein consumers hope—without proof—that systems are able to process their waste; a mismatch between aspiration and reality often causing more harm than good.

An estimated 172 million tonnes of plastic (raw materials and products) were imported into the African continent between 1990 and 2017,^67^ where the average rate of inadequately managed waste is estimated to be around 74%.^14^ In these circumstances, it is logical that food and beverage plastic packaging largely ends up as pollution, with human impacts.^68^ That Unilever uses more plastic per unit of sales in low-income and middle-income countries than in high-income countries only makes matters worse.^61^ Without the unsafe work of informal waste pickers—quite literally picking up the pieces of Big Food —the extent of this pollution would be far greater.^69^

With a history like this, it is not surprising that new initiatives such as the *Plastics Recycling Alliance*—launched in 2019 by Coca Cola, PepsiCo, Nestlé among others, to promote public–private partnerships to improve recycling capacities across Africa—are met with scepticism.^70 71^

There is no doubt that action on plastic is rapidly increasing, with the Big Food actors mentioned here moving faster than ever before as the era of extended producer responsibility becomes inevitable. However, in the face of clear evidence, a history of feet-dragging, regulatory interference and obfuscating does not engender trust. If the plastics crisis highlights anything, is it the interdependencies among industries like Big Food, Big Plastic and Big Oil.

## Conclusion

The case studies presented in boxes 1 and 2 are illustrative examples, among many, of Big Food’s track record which demonstrate a lack of responsibility that, in turn, fails to engender trust. Given this track record, we ask: is it reasonable to expect researchers, practitioners and government policy-makers to engage with such companies in processes of evidence generation, policy agenda-setting or determining solutions for food systems transformation? And if so, where should the onus for such engagement lie? Our stance aligns broadly with the last position presented by Stuckler and Nestle^33^—that powerful transnational companies must demonstrate clear adherence to the principle of ‘do no harm’ and make significant, independently verifiable progress in improving food environments and impacts along product life-cycles before mutual engagement and collaboration can be expected. The ‘walk’ should precede the ‘talk’ if trust is to be earned…not the other way around. This means that robust systems of accountability must be put in place and sustained to meaningfully monitor progress across geographies and domains, and track impacts generated at all stages of a product’s life-cycle.^52^

If food system transformation is to be more than a passing narrative, then the inherent risks of research and policy engagement with Big Food must be taken seriously and navigated with care. Recent events—such as the UNFSS—suggest that this view is not shared by those promoting multistakeholder models of participation open to anyone and everyone—regardless of who they are, what their motives are, and what power they hold. Implicit in this model are at least two assumptions. First, that Big Food can be persuaded to do the right thing for human and planetary health by being present at decision-making tables, while not overshadowing or excluding other less-powerful stakeholders. And second, that this involvement does not confer legitimacy or acceptability on Big Food’s harmful products and practices among international communities of public and planetary health. We question both of these assumptions. First, because they imply that more information and collaboration will somehow provoke companies into fundamentally changing their business practices and ethics, as opposed to simply reformulating products—an ideal largely unsupported by history. And second, because unconditionally opening the door to Big Food jeopardises the integrity of research and policy-making processes while simultaneously implicitly (if not explicitly) conferring legitimacy by becoming part of the ‘food and nutrition community’.

These concerns relate to a simple question: Should Big Food be an external follower of rules, regulations and laws designed by governments to protect people and planet from commercial harm? Or should Big Food be helping to formulate these very protections? We argue for the former, on the basis that the latter equates to a fundamental conflict of interest resulting in fatal weaknesses among food systems ‘solutions’. The latter appears to apply a commercial determinants of health model to locate responsibility but then uses this information in a flawed manner—to bring these actors into solution-determining processes rather than to obtain insights at arms-length.

While we support the optimistic notion that trust is not immutable,^43^ as long as powerful multinational companies are driven primarily by profit maximisation and cannot demonstrate authentic transparency, the likelihood of trust being built and maintained appears unrealistic, if not naïve and illogical to expect. If this remains the case, then we agree with Sula-Raxhimi *et al*^35^ that ‘to improve planetary health and produce healthy livelihoods around the world, solutions must be sought outside the wealth logic mechanisms of corporations.’

## Data Availability

No data were collected for this article.
